# Exploring the Pharmacological Potential of *Lithospermum officinale* L.: A Review of Phytochemicals and Ethnomedicinal Uses

**DOI:** 10.3390/molecules29081856

**Published:** 2024-04-19

**Authors:** Gulzhanat Barkizatova, Aknur Turgumbayeva, Kairat Zhakipbekov, Kuralay Bekesheva, Zhalgaskali Arystanov, Tanagul Arystanova, Farida Kayupova, Klara Zhumalina, Zhanat Toxanbayeva, Aigul Ibragimova, Olga Blinova, Gulnara Utegenova, Nurzhan Iztileu, Zhanserik Shynykul

**Affiliations:** 1 School of Pharmacy, S.D. Asfendiyarov Kazakh National Medical University, Tole Bi St. 94, Almaty 050000, Kazakhstan; b.g_kz@mail.ru; 2 Higher School of Medicine, Al-Farabi Kazakh National University, Almaty 050040, Kazakhstan; 3 Department of Organization, Management and Economics of Pharmacy and Clinical Pharmacy, Asfendiyarov Kazakh National Medical University, Tole Bi St. 94, Almaty 050000, Kazakhstan; zhakipbekov.k@kaznmu.kz; 4 JSC “Scientific Centre for Anti-Infectious Drug”, Astana 010000, Kazakhstan; kuralayaryn02@gmail.com; 5 Department of Pharmaceutical Disciplines, Astana Medical University, Beibitshilik Street 49/A, Astana 010000, Kazakhstan; arystanov.zh@amu.kz (Z.A.); tanagul@mail.ru (T.A.); iztileu.n@amu.kz (N.I.); 6 Department of Pharmacy, Kazakh-Russian Medical University, Abylai Khan St. 51/53, Almaty 050004, Kazakhstan; farida_temir@mail.ru (F.K.); zhumalinaa1962@mail.ru (K.Z.); 7 Department of Pharmacology, Pharmacotherapy and Clinical Pharmacology, South Kazakhstan Medical Academy, Al Farabi Sq. 1, Shymkent 160019, Kazakhstan; zhanat_2701@mail.ru (Z.T.); gup1.pharmacy@gmail.com (A.I.); 8 Department of Organization and Management of Pharmaceutical Business, South Kazakhstan Medical Academy, Alfarabi Sq. 1, Shymkent 160000, Kazakhstan; blinova67@mail.ru (O.B.); gulnara64.64@mail.ru (G.U.)

**Keywords:** *Lithospermum officinale* L., phytochemicals, ethnomedicine, medicinal plants, biodiversity, alkaloids, phenolics

## Abstract

Exploring phytochemicals from ethnomedicinal plants for pharmacological applications is a promising research area. By studying ethnomedicine, researchers can identify plants used for centuries to treat ailments and investigate their phytochemicals. Consequently, phytochemicals can be isolated, characterized, and tested for pharmacological activities, leading to new drug development. This research also helps preserve traditional knowledge and biodiversity. *Lithospermum officinale* L., found in Eurasia, Argentina (South), Colombia, and the United States, is valued for its medicinal properties, including anti-inflammatory, antioxidant, and antimicrobial effects. The current review emphasizes *L. officinale* L. as a significant reservoir of bioactive phytochemicals, with alkaloids, quinones, glucosides, phenolics, flavonoids, and lipids identified as the principal metabolites. It also unveils the unexplored potential of this plant for future research endeavors. Continued research on *L. officinale* L. can unlock its full potential, providing insights into its medicinal uses and contributing to biodiversity preservation.

## 1. Introduction

Exploring phytochemicals from plant species for pharmacological applications based on ethnomedicine is a promising area of research [1]. Ethnomedicine, which involves the traditional medicinal practices of various cultures, offers a wealth of knowledge about the healing properties of plants [2]. Phytochemicals, the biologically active compounds found in plants, have shown great potential in pharmacology. By studying ethnomedicine, researchers can identify plants that have been used for centuries to treat ailments and investigate the phytochemicals responsible for their medicinal properties [3]. These phytochemicals can then be isolated, characterized, and tested for their pharmacological activities [4]. This research can lead to the development of new drugs or therapeutic agents for various medical conditions [5]. Moreover, exploring phytochemicals from ethnomedicinal plants can also help in preserving traditional knowledge and biodiversity [6]. Overall, the exploration of phytochemicals from plants based on ethnomedicine holds great promise for advancing pharmacological research and drug discovery [7].

*Lithospermum*, a genus within this family, comprises about 50 to 60 species, including *L. officinale* L., native to Eurasia but also found in Argentina (South), Colombia, and the United States [8,9,10]. *L. officinale* L. is a valuable plant source due to its medicinal properties. It has been traditionally used in ethnomedicine for its anti-inflammatory, antioxidant, and antimicrobial effects. The plant contains various phytochemicals, such as phenolic compounds and flavonoids, which contribute to its therapeutic properties. Additionally, *L. officinale* L. is known for its potential hepatoprotective and neuroprotective effects, making it a subject of interest for pharmaceutical research. Its wide distribution and adaptability to different climates further highlight its importance as a valuable genetic resource. Through continued research and conservation efforts, the full potential of *L. officinale* L. can be unlocked, providing new insights into its medicinal uses and contributing to the preservation of biodiversity [11].

Plants have been fundamental to human existence, providing essential resources like food, shelter, clothing, and medicine throughout history [12,13,14]. Many cultures revere plants as gifts fulfilling various human needs, particularly in less developed countries where approximately 80% of the population relies on traditional medicine for healthcare [15,16]. Medicinal plants, including *L. officinale* L., play a pivotal role in traditional medicine, with over 3.3 billion people in less developed countries regularly using them [17]. These plants have been integrated into various forms of traditional medicine, such as folklore remedies, decoctions, and oils, contributing to the appreciation of plant diversity [18,19,20,21,22].

*L. officinale* L. is a prime example of a medicinal plant used in ethnomedicine across different cultures. This plant has been extensively used in China, India, Spain, Poland, and North America for medicinal purposes. Archaeological findings in China further reveal its decorative use, indicating its cultural significance and versatility throughout history. These examples highlight the profound impact of plants like *L. officinale* L. on human culture and medicine, showcasing their importance in traditional and historical contexts [23,24,25,26].

The study of phytoconstituents in *L officinale* L. is crucial, especially in pharmacology, due to the plant’s rich composition of various beneficial compounds. These include alkaloids, quinones, glucosides, phenolics, flavonoids, and lipids. These natural compounds have diverse pharmacological applications, making them valuable for medical research and drug development. These compounds, abundant in nature, offer a promising avenue for developing novel therapeutics with a wide range of pharmacological applications. Understanding and harnessing the pharmacological potential of phytoconstituents in *L. officinale* L. could lead to the development of new medicines to treat various diseases. The purpose of this review was to offer comprehensive and informative details regarding the botanical description, geographical distribution, traditional uses, phytochemistry, and pharmacology of *L. officinale* L.

## 2. Methods

A comprehensive search of scientific databases, including Google Scholar and PubMed, was conducted. The search included keywords such as “*L. officinale* L.”, “*L. officinale* L. compounds”, “*L. officinale* L. phytochemicals”, “*L. officinale* L. pharmacological”, and “*L. officinale* L. traditional uses”. The number of search results for each keyword is summarized in Table 1. The selection criteria for articles included studies on the traditional uses, phytochemistry, and pharmacological properties of the *L. officinale* L. genus. Additional studies were identified through a manual screening of the references in the selected articles. Books with high-quality taxonomic and ethnobotanical information were also reviewed. Data collection spanned from 1952 to 2023.

## 3. Distribution and Botanical Characterization

**Figure 1:**
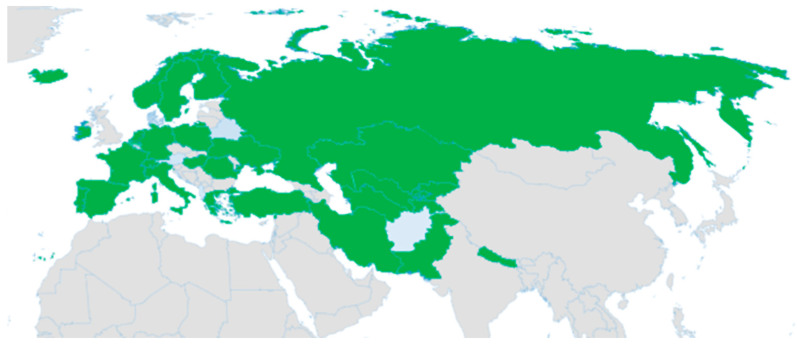
The distribution of *L. officinale* L. using shaded green color [11].

The Boraginaceae family is a diverse group of plants, encompassing approximately 135 genera and 2600 species worldwide [8]. The wide distribution and diverse characteristics of the Boraginaceae family highlight its importance in ecosystems and human culture. These plants are found in various climates, ranging from tropical to temperate regions. One genus within this family is *Lithospermum*, which includes about 50 to 60 species. Among these species is *L. officinale* L., a plant native to Eurasia (Figure 1). Moreover, it has been reported that *L. officinale* L. has been introduced into Argentina (South), Colombia, and the United States [9,10,11]. *L. officinale* L. is a perennial plant that thrives primarily in temperate biomes. Without further research and conservation efforts, it is not possible to uncover the potential benefits of plants like *L. officinale* L. and preserve their biodiversity for future generations.

*L. officinale* L. typically grows up to 90 cm tall. Its stem is usually branched and covered with both subappressed antrorse (forward-pointing) and patent (outward-pointing) hairs, which can be up to 1.5 mm long and have a swollen base. The leaves are lanceolate or broadly so, measuring about 60–70 × 10–16 mm, and are covered with antrorse hairs that are approximately 1.9 mm long, also arising from a swollen base. The flowers of *Lithospermum officinale* L. are arranged in a racemose manner and are bracteate (bracts) or subsessile (almost sessile or without a distinct stalk). The bracts are leafy but smaller than the leaves. The pedicel (the stalk of a single flower in a cluster) is pubescent and can be up to 4 mm long in fruit [27,28].

The calyx (the outer whorl of a flower, typically green and leaf-like) of *L. officinale* L. is 4–5 mm long, antrorsely hairy, and slightly longer in fruit, with linear lobes. The corolla (the petals of a flower, collectively) is white, with a tube that is approximately 4 mm long. The lobes of the corolla are spread out, ovate–obtuse, and crenulate–wavy, with a limb that is 3.5–4 mm broad. The throat of the corolla has five sac-like pubescent pouches. The anthers (the pollen-producing part of a flower) are oblong, about 1 mm long, and situated below and alternating with the pouches, subsessile, and apiculate. The style is 1.7 mm long, with a sub-capitate stigma. The nutlets (small, hard, one-seeded fruit) are 3–4 mm long, ovoid, pale white, smooth, and shiny [28].

## 4. Historical and Cultural Uses of *L. officinale* L.

Plants constitute one of the fundamental kingdoms of life forms. Throughout human history, plants have served as a vital source for essential needs such as food, shelter, clothing, and medicine [29]. Many cultures hold plants in high regard, viewing them as gifts intended to fulfill mankind’s dietary, medicinal, and other requirements. It is estimated that approximately 80% of the global population, particularly in less developed countries, relies heavily on traditional medicine for primary healthcare, according to the World Health Organization. Medicinal plants form the cornerstone of traditional medicine, with over 3.3 billion people in less developed countries utilizing them regularly [30]. Over the course of history, various forms of traditional medicine, including folklore remedies, decoctions, and oils, have incorporated plants as key sources of medicine. This ongoing utilization of plants has not only provided new remedies but has also contributed to the human race’s appreciation of the unique structural diversity found in plant species [31].

One of the best examples of medicinal plants which are used in ethnomedicine in various cultures is *L. officinale* L. [32,33]. Ancient records reveal a rich history of the medicinal and decorative uses of *L. officinale* L. The fruits of this plant were reportedly used as an antiseptic in Poland during a period spanning from about 1750 to 1600 B.C. This highlights the early recognition of its medicinal properties. In various parts of the world, including China, India, Spain, and North America, ancient ethnomedicine documents the use of different parts of *L. officinale* L. to treat a variety of illnesses, showcasing its widespread recognition as a medicinal plant across diverse cultures.

Moreover, archaeological findings in the Yanghai Tombs (Xinjiang, China) provided evidence of the plant’s decorative use. Fruits of *L. officinale* L. were discovered adhered to two wooden tubs, indicating that they were used as a form of decoration. This dual application of the plant, both as a medicinal agent and as a decorative element, underscores its cultural significance and the versatility of its uses throughout history (Table 2).

The historical applications of *L. officinale* L. underscore its enduring value as a plant source. These findings highlight the plant’s extensive history of use across different cultures and time periods. To fully appreciate its value, it is essential to explore the phytoconstituents present in all parts of *L. officinale* L.

## 5. Phytochemistry

Studying the plant phytoconstituents of *L. officinale* L. is important for several reasons, particularly in terms of pharmacology. *L. officinale* L. contains a group of phytochemicals, including alkaloids, quinones, glucosides, phenolics, flavonoids, flavonol glycosides, and lipids, which possess a wide range of pharmacological activities (Table 3). For instance, while quinones, known for their antioxidant and anti-inflammatory effects, are also studied for their antimicrobial and anticancer activities, glucosides can act as antioxidants, anti-inflammatories, or exhibit other specific pharmacological effects [39,40,41]. Moreover, phenolics, such as phenolic acids and flavonoids, are renowned for their antioxidant and anti-inflammatory properties, with potential benefits against cancer, diabetes, and neurodegenerative diseases [42,43,44]. Additionally, flavonoids, a subclass of phenolics, demonstrate a wide range of pharmacological activities, including antioxidant, anti-inflammatory, anticancer, and cardiovascular protective effects [45]. Flavonol glycosides share similar properties and are particularly studied for their antioxidant and anti-inflammatory effects [46]. In addition, lipids exhibit anti-inflammatory, anticancer, and cardiovascular protective effects [47]. These compounds, found abundantly in nature, offer a promising avenue for the development of novel therapeutics.

## 6. Possible Pharmacological Activities of Compounds

### 6.1. Alkaloids

Among the found alkaloids, allantoin possesses antinociceptive, anti-inflammatory, wound healing, and keratolytic effects [49,50,51]. The study on the wound healing process found that it accelerates healing by regulating inflammation and stimulating fibroblast proliferation and extracellular matrix synthesis. As a result, allantoin was shown to enhance and expedite the restoration of normal skin [81]. Another study evaluated the antinociceptive and anti-inflammatory effects of allantoin, and it was found that the administration of allantoin at a dose of 60 mg/kg demonstrated significant anti-inflammatory activity in a carrageenan-induced paw edema model. Additionally, allantoin reduced leukocyte migration and pleural exudate in the pleural cavity [82].

However, the literature analysis did not provide any information about the mechanisms of action and potential therapeutic benefits of lithosenine and acetyllithosenine. It was concluded that more research is needed to fully understand their mechanisms of action and potential therapeutic benefits. In addition, 07-3-hydroxy-3-methylbutanoyl-09-(-)-hydroxyviridifloryl retronecin and its acetyl derivative, isolated from *L. officinale* L., have not been studied for their pharmacological activities, and their properties remain unknown.

Another two alkaloids, lycopsamine and echimidine, were found to be toxic and potentially carcinogenic due to their hepatotoxicity [54]. However, the plant cannot be omitted from the roster of medicinal plants due to the fact that the specific plant parts where these compounds amass remain undetermined, thus hindering the acquisition of a beneficial extract. Additionally, it has been documented that pyrrolizidine alkaloids (such as lycopsamine and echimidine) exhibit poor absorption rates via the human dermis [83], thereby enabling the utilization of the derived extracts containing diverse biological metabolites (such as lycopsamine and echimidine) in the form of an antibacterial ointment.

### 6.2. Quinones

Alkannin and shikonin are naphthoquinones that occur naturally and are predominantly found in plants of the Boraginaceae family [33]. Both compounds have a wide range of pharmacological applications. For example, their antitumor activity involves apoptosis, necroptosis, and immunogenic cell death. This activity is related to the naphthoquinone scaffold’s ability to generate reactive oxygen species (ROS) and act as an alkylating agent. The antitumor mechanisms of naturally occurring shikonin, alkannin, and their derivatives include direct interactions such as covalently binding to DNA and proteins like alkylating agents, as well as indirect interactions mediated by ROS, which nonspecifically influence mitochondria or multiple signal pathways [84]. Moreover, they are recognized for their anti-inflammatory, antimicrobial, antioxidant, wound-healing, and anticancer properties [56,58,59,60].

### 6.3. Glucosides

A new glucoside, 6-O-β-D-glucopyranosyl-1-cyanomethylene-4,5-dihydroxy-2-cyclohexene, has been isolated from the roots of *L. officinale* L. [85]. However, there is no information available regarding the pharmacological activity of 6-O-β-D-glucopyranosyl-1-cyanomethylene-4,5-dihydroxy-2-cyclohexene.

### 6.4. Phenolics

Rosmarinic acid is a phenolic compound with bioactive properties that is commonly present in plants belonging to the Lamiaceae and Boraginaceae families. Numerous scientific papers suggest that rosmarinic acid has potential as an antimalarial, antiviral, and antibacterial agent. Additionally, its strong antioxidant properties have recently made it a focus for potential use as a nutraceutical compound in the food industry [86]. Rosmarinic acid demonstrates its anticancer activity through various mechanisms. It induces apoptosis in prostate cancer cells by modulating intrinsic mitochondrial apoptotic pathway mediators. Additionally, it inhibits proliferation and invasion in hepatocellular carcinoma cells by targeting the *PI3K/Akt/mTOR* signaling pathway. Moreover, rosmarinic acid blocks *FOXOM1* transcription factors, upregulates pro-apoptotic genes, and exhibits antitumorigenic actions in triple-negative breast cancer cells. Furthermore, it has reduced pancreatic ductal adenocarcinoma by inducing G1/S cycle arrest and inhibiting Gli translocation in a mouse model of PDAC [63,64,85].

### 6.5. Flavonoids and Flavonol Glucosides

Hydroxylated polyphenols, known as flavonoids, are abundant in various plant sources like vegetables, fruits, cereals, nuts, herbs, seeds, stems, and flowers. These compounds exhibit various medicinal properties, including antioxidant, anticancer, antimicrobial, neuroprotective, and anti-inflammatory effects [87]. An exemplary flavonoid present in *L. officinale* L. is luteolin-7 beta-glucuronide, known for its antioxidant, anti-inflammatory, antiallergic, neuroprotective, and cardio-protective properties [68,69,70].

Rutin, a flavonoid present in numerous plants, exhibits diverse biological properties, such as anti-inflammatory, antioxidant, neuroprotective, nephroprotective, and hepatoprotective effects [72]. Moreover, the antihyperglycemic property of rutin and its protective effects against diabetic complications have been discussed, with proposed mechanisms including reduced carbohydrate absorption from the small intestine, the inhibition of tissue gluconeogenesis, increased tissue glucose uptake, the stimulation of insulin secretion from beta cells, and the protection of Langerhans islets from degeneration. Rutin also reduces the formation of sorbitol, reactive oxygen species, advanced glycation end-product precursors, and inflammatory cytokines. These effects are thought to underlie rutin’s protection against nephropathy, neuropathy, liver damage, and cardiovascular disorders induced by hyperglycemia and dyslipidemia [88].

### 6.6. Lipids

Lipids, particularly fatty acids, are another group of major constituents found in *L. officinale* L. with significant biological activities. Among these, γ-linolenic acid (GLA) stands out for its diverse benefits. GLA is known for its anti-inflammatory properties, making it valuable in managing conditions related to inflammation. It also plays a role in promoting skin health, as it contributes to the maintenance of the skin’s natural barrier function and hydration. Additionally, GLA is involved in hormonal balance, particularly in women, and supports the immune system, aiding in its proper functioning [74,75,76].

Another notable fatty acid found in *L. officinale* L. is stearidonic acid (SDA), which shares some similarities with GLA in terms of its beneficial effects. Like GLA, SDA exhibits anti-inflammatory properties, making it potentially useful in managing inflammatory conditions. It also contributes to skin health by supporting the skin’s barrier function and hydration levels. Moreover, SDA is involved in hormonal balance and supports the immune system, further highlighting the diverse biological activities of lipids found in *L. officinale* L. [77,78].

Δ5-avenasterol is a phytosterol, a type of plant sterol, that is found in various plant sources, including vegetables, fruits, nuts, and seeds. It is particularly abundant in cereal grains, such as oats and wheat. Δ5-avenasterol has been studied for its potential health benefits, including its cholesterol-lowering effects. Research suggests that Δ5-avenasterol may help lower cholesterol levels by inhibiting the absorption of dietary cholesterol in the intestine. It is believed to compete with cholesterol for absorption, thereby reducing the amount of cholesterol that enters the bloodstream. By lowering cholesterol levels, Δ5-avenasterol may help reduce the risk of cardiovascular diseases, such as heart disease and stroke. In addition to its cholesterol-lowering effects, Δ5-avenasterol has also been studied for its anti-inflammatory properties. Some studies suggest that it may help reduce inflammation in the body, which is a key factor in the development of various chronic diseases [79,80].

## 7. Pharmacological Effects Studies on *L. officinale* L.

*L. officinale* L. has been the subject of some pharmacological studies due to its rich composition of bioactive compounds. For instance, the neuroprotective effect of *L. officinale* L. callus extract (LoE) on inflamed primary microglial cells was investigated. LoE, derived from the fresh cells of *L. officinale* L., was evaluated for its anti-inflammatory capacity on rat microglial cells, which are crucial in responding to neuroinflammation. The results showed that the methanolic extract of the 17-day-old callus of *L. officinale* L. exhibited significantly higher anti-inflammatory effects on lipopolysaccharide (LPS)-stimulated microglial cells compared to commercial formulation A (CfA). This was supported by reduced expression of inflammatory markers (*Nos2*, *Tnf-α*, *Cox-2* mRNA) and suppression of TNF-α and IL-1β release in activated microglial cells treated with an effective dose of LoE (0.8 mg/mL). Moreover, the study suggests that the superior anti-neuroinflammatory performance of LoE compared to CfA in LPS-activated primary microglia may be due to the synergistic effects of its components and the lipophilic nature of rosmarinic acid, the main phenolic acid in LoE [89]. Since LoE has a high antioxidant capacity and could be a reliable substitute for the preparation of neuroprotective pharmaceutical formulations, further in vivo research and experiments are needed to confirm these findings.

Another study compared the effects of *L. officinale* L., silver sulfadiazine (SSD), and alpha ointments on burn wound healing in rats. The results showed that *L. officinale* L. and SSD application decreased the number of inflammatory cells, with *L. officinale* L. being more effective than alpha ointment. The frequency of macrophages decreased after burn injury, but the decrease was most significant with *L. officinale* L. and alpha ointment. Re-epithelialization, angiogenesis, and granulation tissue formation were best with *L. officinale* L. and alpha ointment, while the worst results were seen in the burn injury group and SSD group regarding granulation tissue formation. A histological assessment revealed that *L. officinale* L. and alpha ointment were most effective in reducing inflammation, promoting re-epithelialization, angiogenesis, granulation tissue formation, and reducing macrophage numbers after burn injury [90].

Moreover, the study investigated the antithyrotropic activity of freeze-dried extracts from *L. officinale* L. (Lith. off. FDE) in rats and compared its effects with those of potassium iodide (KI). When Lith. off. FDE was administered with thyroid-stimulating hormone (TSH), it blocked the TSH-induced increase in endocytotic activity of the thyroid glands, leading to a significant decrease in thyroid hormone levels. When administered alone, Lith. off. FDE caused a decline in endogenous TSH levels, thyroidal secretion, and thyroid hormone levels. A comparative analysis with KI showed that Lith. off. FDE had a more rapid onset and longer duration of action in blocking thyroid secretion, suggesting a different mode of action. The study also demonstrated that Lith. off. FDE inhibited peripheral T4-deiodination in thyroidectomized and T4-substituted rats. Overall, the findings suggest that Lith. officinale extract may have potential as a therapeutic agent for thyroid disorders, and further research is warranted to elucidate its specific mechanisms of action and clinical implications [91,92].

## 8. Conclusions

*L. officinale* L. has a rich history of use in various cultures. Significant research on its phytoconstituents and pharmacological activities was conducted between 1975 and 2010. However, in the last 15 years, there has been very limited research dedicated to the analysis of its phytoconstituents and the evaluation of its pharmacological activities. This could be due to a variety of factors, such as shifting research priorities, limited funding, or the complexity of studying these compounds. It is important to acknowledge the value of older sources that contain significant information but also to recognize the need for updated research to further our understanding of this plant’s potential uses and safety profile.

The literature review analysis revealed the presence of various categories of substances with recognized pharmacological properties. However, the presence of toxic pyrrolizidine alkaloids, such as lycopsamine and echimidine, possibly limits its use. Further research is needed to determine which parts of the plant accumulate these compounds and to explore safe ways to utilize its beneficial components, such as incorporating them into topical treatments or developing controlled-release formulations.

## Figures and Tables

**Table 1:** The number of searches for each keyword.

Keywords	Number of Searches	
	Google Scholar	PubMed
*L. officinale* L.	13,100	48
*L. officinale* L. compounds	3210	3
*L. officinale* L. phytochemicals	1560	2
*L. officinale* L. pharmacological	2840	6
*L. officinale* L. traditional uses	4910	—

“—” means not found.

**Table 2:** Ethnomedicinal use of *L. officinale* L. in various cultures.

Country	Plant Part	Ethnomedicinal Use(s)	Findings	References
Poland	fruit	nuts of *L. officinale* L. were utilized as an antiseptic	1. from about 1750 to 1600 B.C.; 2. a plaster made of tar with the fruit of *L. officinale* L.	[34]
China	fruit	1. a treatment for urogenital tract disorders and as a medication for relaxing spasms; 2. utilized as an ancient form of plant adornment	1. 2500 years BP; 2. fruit from *L. officinale* L. were found adhered to two wooden tubs in the Yanghai Tombs of Xinjiang, China.	[35,36]
North America (by Indians)	root	1. antidiarrhoeal drug; 2. oral contraceptives made from cold water extracts of the root.	saline extract of root	[37,38]
India, Spain	leaves	used as sedative		
India	seeds	used as diuretic and lithotriptic		
India	roots and twigs	utilized as a remedy, a decoction of roots and twigs was administered as a syrup for eruptive diseases like smallpox and measles.		

**Table 3:** Biologically active compounds found in *L. officinale* L.

Chemical Class	Compound	Structure	Known Pharmacological Activities
Alkaloids (pyrrolizidine alkaloids)	allantoin [48]	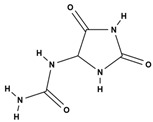	antinociceptive and anti-inflammatory [49], wound healing [50], keratolytic [51]
	lithosenine [52]	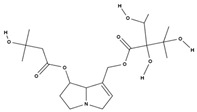	—
	acetyllithosenine [52]	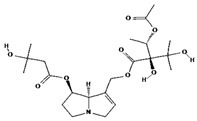	—
	lycopsamine [53]	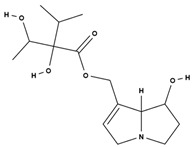	toxic and potentially carcinogenic due to its hepatotoxicity [54]
	echimidine [53]	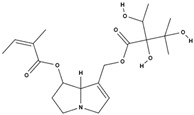	toxic and potentially carcinogenic due to its hepatotoxicity [54]
Quinones (naphthoquinones)	alkannin [55]	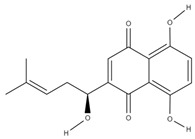	anti-inflammatory, antimicrobial, antioxidant, wound healing, anticancer [56]
	shikonin [57]	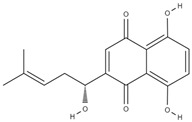	anti-inflammatory, antioxidant, antimicrobial, wound healing, anticancer, antidiabetic, antiallergic [58,59,60]
Phenolics (phenolic acids)	rosmarinic acid [48,61,62]	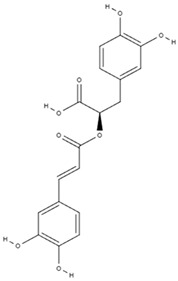	anticancer [63], anti-inflammation, antioxidation, antidiabetes, antivirus, antitumor, neuroprotection, hepatoprotection [64]
	lithospermic acid [65]	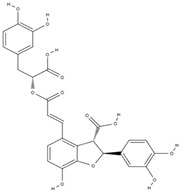	antioxidant, anti-inflammatory, antimicrobial, anticancer, cardioprotective, neuroprotective [66]
	caffeic acid [38]	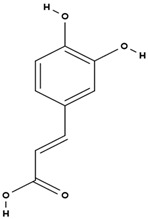	antioxidant, anti-inflammatory, antimicrobial, anticancer (inhibits), neuroprotective, cardioprotective [67]
	chlorogenic acid [38]	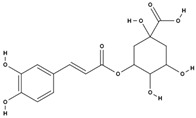	antioxidant, anti-inflammatory, weight loss, antidiabetic, neuroprotective, cardioprotective [67]
Flavonoids	luteolin-7 beta-glucuronide [38]	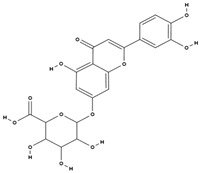	antioxidant, anti-inflammatory, antiallergic, neuroprotective, cardioprotective [68,69,70]
Flavonoids	rutin [71]	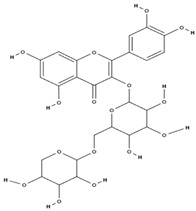	antioxidant, anti-inflammatory, antidiabetic, vasoprotective, neuroprotective, cardioprotective [72]
Lipids (fatty acids)	γ-linolenic acid [73]	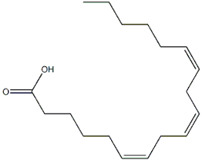	anti-inflammatory, skin health, hormonal balance, immune system support [74,75,76]
	stearidonic acid [73]	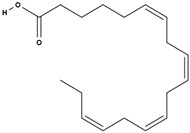	anti-inflammatory, skin health, hormonal balance, immune system support [77,78]
	Δ^5^-avenasterol [73]	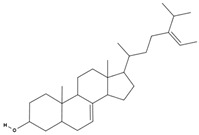	cholesterol-lowering, anti-inflammatory, immune modulation, anticancer, skin health [79,80]

## Data Availability

Data are contained within the article.
